# Are Traditional Registries Becoming Obsolete in the Modern Digital Health Ecosystem?

**DOI:** 10.2196/92696

**Published:** 2026-07-03

**Authors:** Judith Wenk, Hernan Inojosa, Isabel Voigt, Stephen Gilbert, Tjalf Ziemssen

**Affiliations:** 1 Center of Clinical Neuroscience, Department of Neurology Faculty of Medicine and University Hospital Carl Gustav Carus Dresden TUD Dresden University of Technology Dresden, Saxony Germany; 2 Centre for Tactile Internet with Human-in-the-Loop (CeTI), TUD Dresden University of Technology Dresden, Saxony Germany; 3 Else Kröner Fresenius Center for Digital Health, TUD Dresden University of Technology Dresden, Saxony Germany; 4 Faculty of Business and Economics TUD Dresden University of Technology Dresden, Saxony Germany

**Keywords:** digital health, digital twins, EHDS, health data, patient registry, TEFCA

## Abstract

Registries have long been a cornerstone of medical research and public health, providing systematically collected data on diseases, treatments, and health outcomes. However, in the era of digital health, we argue that the traditional model of stand-alone registries needs reconsideration, given the context of increasingly digitized and interoperable health data ecosystems. Unless registries evolve to embrace embedded, standards-based data services, operating across interoperable infrastructure, they will become obsolete while digitalization is reshaping how data can be collected, shared, and used. In this viewpoint, we recount how the present health data ecosystem came to be and what role registries have come to play therein. Following that, we show how recent regulatory initiatives such as the Trusted Exchange Framework and Common Agreement in the United States or the European Health Data Space Regulation signal a shift toward cross-network health information exchange, promoting patient-centric data integration within electronic health record systems. We further illustrate how electronic health records are consequently set to evolve into information hubs, acting as the primary gateway for individuals through which they may access and control their personal health data spread throughout increasingly connected health data ecosystems. This, in turn, might stimulate the creation of digital twins and continuous learning health systems in practice. Following this line of thought, we discuss the opportunities and challenges of interconnected health data ecosystems. Ultimately, we propose that next-generation registries need to be designed as dynamic, service-oriented software stacks for research, leveraging the common data infrastructures that are currently being established around the world. Given the points raised in this viewpoint, we invite health care professionals and researchers alike to equally rethink the role that registries should play within the globally emerging interconnected health data ecosystems and contribute their findings. References included in this viewpoint were identified through searches of PubMed and Google Scholar with various search terms and combinations thereof pertinent to the topics touched on, for example, “patient registry,” “clinical registry,” “digital twin,” “healthcare,” “clinical research,” “virtual twin,” “TEFCA,” or “EHDS.” Only papers in English were reviewed. The final reference list was generated on the basis of originality and relevance to the broad scope of topics covered in this viewpoint, aiming to present a balanced overview of topic-related findings and arguments.

## Introduction

Registries have been instrumental in medical research for over 150 years [1]. By design, all registries share a common feature: they systematically collect uniform data on predefined populations to address specific clinical, epidemiological, or operational questions [2]. Analysts use registry data, for instance, to track the natural history of diseases, evaluate treatment effectiveness, monitor safety, improve quality of care, or conduct public health surveillance [3]. In the predigital era and well into the late 20th century, registries primarily relied on de novo data entry dedicated to the registry’s purpose, frequently in parallel with routine clinical record-keeping.

Over the past 2 decades, patient registries have gained renewed prominence as a key source of real-world data (RWD) for medical and health research [4]. Even though multiple, slightly differing definitions exist [5], data at present commonly qualify as RWD if their collection is not subject to any rules or conditions outside the standard care setting [5-7]. For example, the US Food and Drug Administration broadly defines RWD as “data relating to patient health status and/or the delivery of health care routinely collected from a variety of sources,” and considers real-world evidence to be the clinical insights derived from analysis of RWD.

RWD use has long been advocated in medical research to bridge the efficacy-effectiveness gap, but has experienced a remarkable surge in recent years [8,9]. The COVID-19 pandemic further underscored the need for timely RWD, as policymakers required rapid evidence for decision-making during the crisis [8]. Health technology assessment (HTA) bodies and regulators now increasingly accept and sometimes even request real-world evidence to inform decisions on the effectiveness, safety, and cost-effectiveness of interventions [10,11].

Notably, HTA bodies regard registries as one of the most important RWD sources. In a survey conducted in 2020, 19 of 22 HTA organizations across 21 countries indicated that they accept registry data for decision-making—the highest acceptance among RWD sources studied [12]. Registries’ structured nature and curated data make them appealing for research. However, the same survey revealed a paradox: although registries were deemed the most acceptable RWD source, respondents—when asked to rank barriers to RWD acceptance on a scale from 1 (most important) to 9 (least important)—noted that registries often lack important variables, limiting their usefulness (average=4.2) [12]. This highlights the fact that registries in practice often still fail to appropriately capture the full spectrum of data needed for today’s health questions. Respondents further identified the lack of necessary data sources (average=3.3) and policy structures/information governance (average=3.5) as the top 2 barriers to using RWD.

Indeed, the volume and variety of health data have exploded in the digital age [13]. Nearly every facet of patient care and daily life can generate health-relevant data, from entries in electronic health records (EHRs) and laboratory results to smartphone sensor readings and billing accounts [14,15]. This wealth of data holds enormous potential for insights, but also challenges the traditional paradigm where registries are established as stand-alone data repositories with a predefined structure of fixed tabular fields, but with no or only a few connections to other data sources in use for data collection. Modern health data, however, goes beyond that and nowadays include unstructured notes, images, device telemetry, and more. Restricting analyses to a registry’s predefined dataset can mean losing granularity and context that reside in these other data modalities [16], which is why the recurrent use of fixed electronic case report forms for collecting data in registries deserves reconsideration. Even more so, considering recent regulatory initiatives such as the US Trusted Exchange Framework and Common Agreement or the European Health Data Space Regulation that have been designed to facilitate the transition from disconnected data sources to a “network of networks” for cross-network health information exchange (HIE).

Given the survey results cited above and based on our own personal experience, we believe that researchers and practitioners urgently need to rethink the role of registries in health care and health research. Other authors have even called for the formalization of “Registry Science” as a new research discipline [17]. This viewpoint aims to synthesize topics and scientific findings surrounding health data use to propose a way forward. It accordingly describes the evolution of health data ecosystems and the role that registries have come to play therein. It outlines how digitalization and associated major changes in legislation and regulation pertaining to health data use in the European Union and the United States are transforming these ecosystems. Although the digital twin (DT) concept as such has been widely discussed in the literature in recent years with regard to health care, existing DT applications in practice remain scarce and limited in scope, as far as we are aware. To our knowledge, existing papers have also not yet discussed the implementation of DTs for health care against the backdrop of the aforementioned recent legislative and regulatory changes. With the publication of this viewpoint, we aim to connect the dots and intend to show why and how recent regulatory changes may stimulate the creation and use of DTs in clinical research and care. Eventually, we discuss the opportunities and challenges of the globally emerging interconnected health data ecosystems, based on a narrative review of pertinent literature. In doing so, we hope to inspire more research that supports the modernization of registries while contributing our own personal vision for their future.

## Health Data Are Everywhere

Once IT entered the health sector, professional health data stakeholders such as health care providers or payers eagerly used it to convert paper records to electronic form and digitized their record-keeping processes, but while doing so, largely replicated old workflows in digital format [18]. True digitalization—meaning redesigning processes to fully exploit digital technology [19]—has long lagged behind [18]. As long as fast, stable, and affordable internet connections were still long out of sight, large-scale connectivity and data sharing enabling efficient secondary use of data were not a priority yet. Neither for users and developers, nor for policymakers and regulators. This has left us with a legacy of noninteroperable systems, each a “lock-in” environment with proprietary designs and architectures tied to specific vendors [20,21].

**Figure 1:**
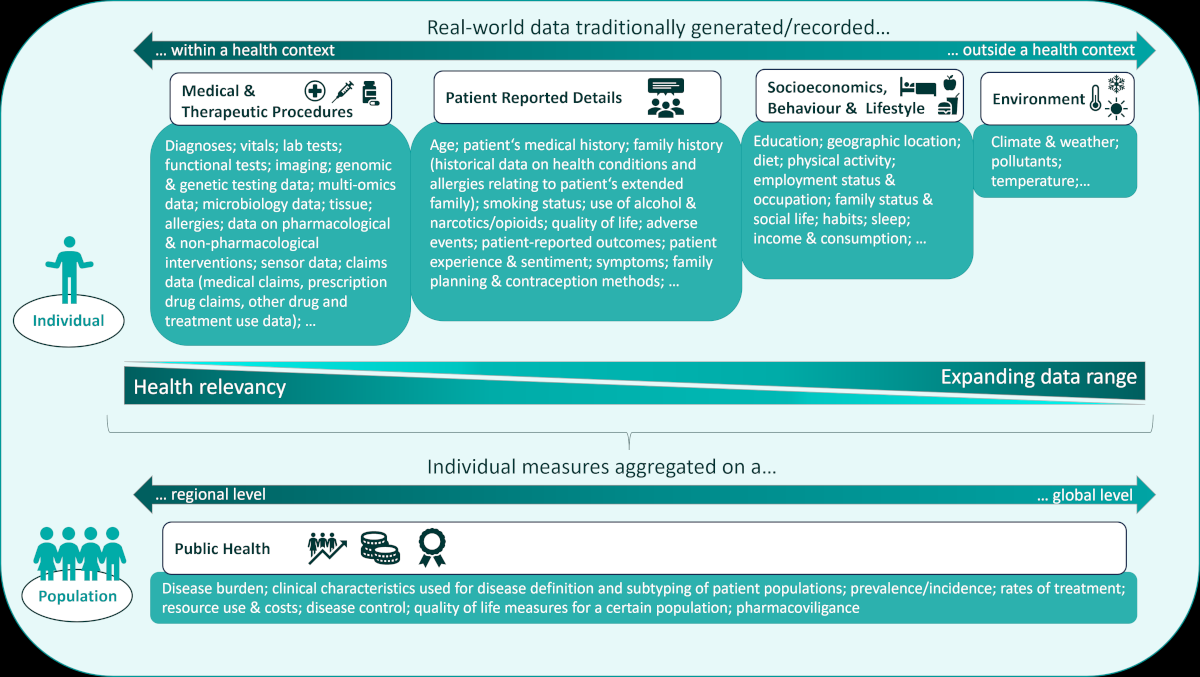
The expanding range of real-world data that potentially bears health-relevant insights. Categories and real-world data examples were adapted from Liu and Panagiotakos, Dang, and Anagnostopoulos et al; the grouping along the continuum within a health context vs outside a health context was inspired by Deloitte. Depiction of health relevancy and expanding data range inspired by Fiske et al. All icons were obtained from the Microsoft 365 Office Suite.

Nowadays, computing power, storage capacity, and network bandwidth are abundant and ever-increasing, marking the maturation and convergence of social, mobile, analytics, and cloud technologies [22]. Global internet and social media use, as well as adoption of mobile and smartphones, continue to grow, passing the 5 billion user threshold of the global population on all accounts today [23]. Meanwhile, the global volume of data is exploding exponentially on the order of zettabytes, and the health sector is a major contributor to this big data revolution [13,15]. Notably, most of these data are RWD [7,24,25], potentially bearing health-relevant insights (Figure 1 [6,7,15,26,27]).

Figure 1 provides a nonexhaustive overview of RWD sources, arranged along a continuum from data typically considered directly health-relevant to data that may become health-relevant when linked with other information. Data may be compiled at the individual or population level depending on the purpose of analysis. For public health reporting, the target population is often defined from a political-geographic perspective, ranging from regional to national to global level data. Other parameters, such as age or income group, can also be used to define the data range and scope for a certain population under investigation.

Not all data are equally helpful for answering health-related questions. For instance, one can distinguish between (1) direct health data, which are generated in the process of health care delivery or health research to answer questions with explicit health relevance (eg, laboratory data), (2) indirect health-relevant data, which are created outside a health care delivery or research context, but that could still be used to answer a question with explicit health relevance (eg, activity tracking via fitness apps), and (3) potentially health-relevant data, which may not seem health-relevant just by themselves, but have the potential to answer a health-relevant question if linked with other data (eg, purchasing data or posts on social media) [27]. Indirect health-relevant data and potentially health-relevant data have received increasing attention in the wake of personalized medicine and precision medicine, fueled by the fast-progressing development and use of artificial intelligence for data-driven health applications worldwide [28-30].

**Figure 2:**
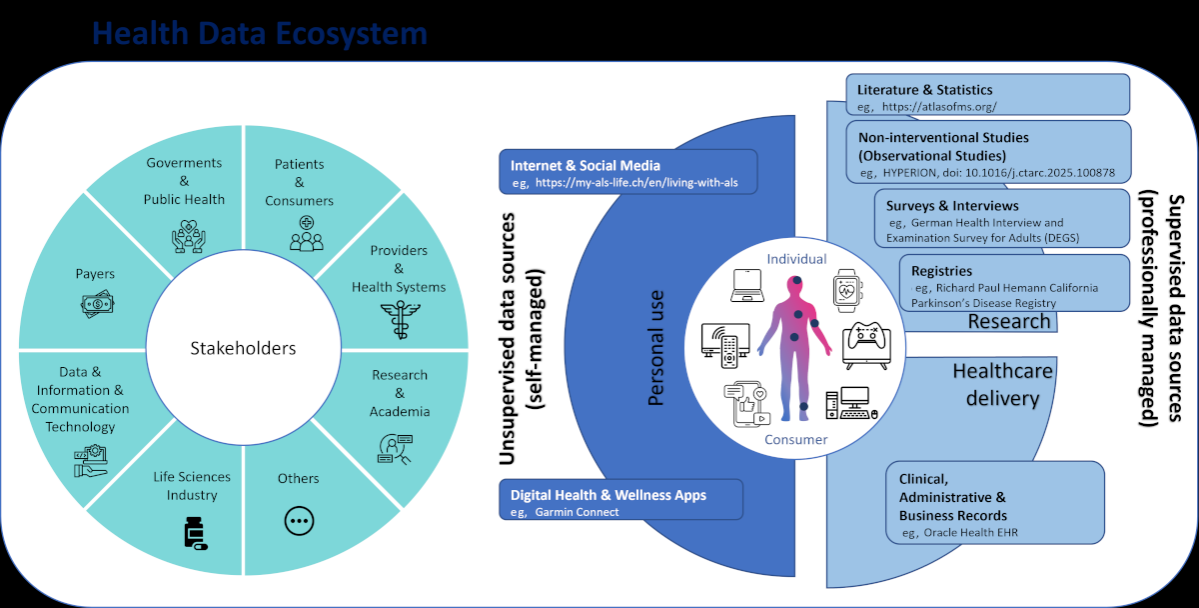
The health data ecosystem. The grouping of stakeholders was adapted from Deloitte and the ONC TEFCATM Recognized Coordinating Entity, and categorization of data sources was adapted from Liu and Panagiotakos, Dang, Graili et al, and Anagnostopoulos et al. Icons made by surang, Uniconlabs, kang somad, SBTS2018, Freepik, DailyPmStudio, inkubators, rukanicon, Nuricon, kmg design, Prosymbols Premium, and IconBaandar from Flaticon [32].

Technological progress has not only considerably expanded the range of RWD that can be deemed health-relevant but has also increased the number of instances in which data are generated in either a personal or professional use context (Figure 2 [6,7,15,25,26,31,32]).

In broad terms, professional stakeholders generate/collect, store, and process data either for health research or health care delivery purposes, while private individuals (private end consumers) generate/collect, store, and process data for all kinds of personal purposes, which may be health-related, but do not necessarily have to be. The clear separation between the 2 major professional use purposes in Figure 2 highlights the historic divergent evolution of separate information systems for one purpose or the other [33], which reinforces interoperability issues we are still facing today. Secondary use overall remains a challenge, as consumer or patient-generated data originating from personal use also are not easily accessible for professional use and vice versa. But no matter where the data resides, who holds or owns it, data that can be helpful for answering a health-related question is data that can be linked to a certain individual. The individual is the point of reference, which is why we have placed in the center of all data sources.

Table 1 provides a more detailed characterization of RWD sources, while Textbox 1 extends the overview of source categories depicted in Figure 2.

**Table 1:** Characterization of real-world data sources. Adapted from [25,27,34].

	Unsupervised data sources (self-managed)	Supervised data sources (professionally managed)
Primary use purpose	Personal use, not necessarily health-related. Personal use can include interaction with these sources for health-related reasons, such as posting questions/experiences online, managing doctor’s appointments, or maintaining a healthy lifestyle (eg, by tracking one’s physical activity), but is not limited to such.	Professional, health-related use. In broad terms, data in these sources are explicitly collected and used either for health research or for health care delivery purposes.
Examples for primary use	Private individuals who... track their physical activities in an app; ... post on blogs or social media; ... use an electronic device of their choice to browse the web for a personal purpose; ... play games or watch videos on an online platform; etc.	Academic researchers who lead a study to explore the pathogenesis of a disease, health care professionals who document procedures and outcomes in clinical records for later reference and billing, pharmaceutical companies that commission scientific studies to obtain evidence for market authorization, etc.
Data review/quality check	Data inclusion in these sources is not subject to the supervision of a trained health professional or any kind of collection protocol.	Prior inclusion in these sources is subject to the supervision of a trained health professional. Data usually go through internal review (eg, screening of study candidates by health care professionals before recruitment; examination of a patient for confirmation of diagnosis) or external review (eg, peer-review of scientific publications, study monitors) to ensure that any data meet set requirements and are organized in a fairly standardized manner (eg, following a given study protocol, in compliance with legislative regulation and rules).
Health relevance of data included in these sources	Indirect health-relevant data and potentially health-relevant data	Direct health data

**Comprehensive categorization of real-world data sources. Adapted from [6,7,25,26,34].**

**Unsupervised real-world data (RWD) sources (self-managed)**

- Personal use
  Internet and social media
    Online communities
    Blog and vlogs
    Twitter, Facebook, Instagram, TikTok, YouTube, etc.
  Digital health and wellness apps
    Trackers and diaries (eg, habits and activities, symptoms, and menstruation)
    Motoric and cognitive tests
    Surveys and interviews
    Educational content (eg, videos, quizzes, and information texts)
    Digital apps, however, can be equipped with technical features that allow for the automatic check of data entry. For this reason, digital health apps that have been approved as a medical device or apps whose use is overseen by health care professionals (eg, app use as part of a study) would constitute supervised sources.

**Supervised RWD sources (professionally managed)**

- Research
  Noninterventional studies (observational studies)
    Cross-sectional studies
    Case-control studies
    Cohort studies
    Pragmatic trials
  Surveys and interviews
    Health and patient surveys
    Interviews and focus group discussions
  Registries
    Patient registries
    Disease registries
    Health service registries
  Literature and statistics
    Scientific journals and publications (preferably peer-reviewed)
    Health figures and recommendations published by professional associations, patient organizations, governments, and nongovernmental organizations
- Health care delivery
  Clinical, administrative, and business records
    Electronic health records and medical records
    Insurance claims and billing datasets
    Employment records
    Case notes and medication orders
    Admission/discharge and progress reports
    Medication administration records (dose, route, and National Drug Code/RxNorm codes)
    Point of sale records (prescriptions including refills and over-the-counter drugs, health and wellness goods/services)
    Tax records

In conclusion, present health data ecosystems encompass a vast array of stakeholders that use various digital devices and applications to generate, collect, process, and store more data of varying health-relevance for either personal or professional health-related use purposes than ever before, while health data sharing remains complicated [35-38].

## The Rise of Interoperability Initiatives

There is a growing consensus among stakeholders that data should be collected once, and used many times, provided proper governance and consent are in place (eg, the summary of research survey results from multiple countries in [39] and the scoping review by Alam et al [40]). This requires integrated architectures through which data from multiple sources can be accessed and reused for multiple purposes. Along these lines, the concept of the learning health system (LHS), sometimes more narrowly conceptualized as a learning health care system [41], has gained traction over the past 2 decades, with stakeholders envisioning a cycle where practice and research through large-scale data integration mutually inform each other and thereby induce a cycle of improvement [4,42]. In parallel, demands for greater individual autonomy in terms of data access and control have been articulated [4], accelerating efforts to integrate data collection across platforms.

Over the past decade, many attempts to link EHRs and registries have been made, accompanied by a multitude of studies investigating the extent to which EHR systems could be adopted and used for research purposes [33,43-45]. But any such integration of information systems has typically been tailored to a specific use case or a specific pair of systems again. As a result, the broader interoperability problem has yet to be resolved: numerous custom pipelines have been built, but each is limited in scope (eg, linking 1 hospital’s EHR to 1 specialty registry). HIE takes place over many point-to-point connections rather than an open network.

In the United States, following the introduction of the Health Information Technology for Economic and Clinical Health Act in 2009, numerous vendor-, state-, and consortium-led efforts, while aiming to resolve this, have produced a “patchy landscape” of health information networks with divergent architectures and standards [46]. Other countries, too, have taken actions to transform their HIE infrastructures and processes, but the level of HIE maturity still varies widely, even within countries that already exhibit a comparably high level of digitalization (for an overview of different countries, see [35-38]). More recently, however, 2 regulatory initiatives have attracted particular attention because of their scope and expected impact.

In 2022, the 21st Century Cures Act’s Trusted Exchange Framework and Common Agreement (TEFCA) was introduced to create a nationwide “network of networks” for HIE within the United States. In the same year, the European Health Data Space (EHDS) Regulation was proposed, which likewise sets out a legal and technical framework for cross-border HIE and use of health data across all member states of the European Union [47]. The EHDS entered into force on March 26, 2025. Like TEFCA, it reinforces data portability to ensure individual data access and control, which is crucial for building dynamic, composite EHRs. Table 2 presents a comparison of the core characteristics of both initiatives.

**Table 2:** The US approach vs the European approach to building an infrastructure for health information exchange (HIE), enabling large-scale data integration. Adapted from [31,47].

	TEFCA^a^ [31]	EHDS^b^ [47]
Scope of application	Focuses on the US health system; applies to health information networks that have been designated as Qualified Health Information Networks by the RCE^c^, their participants, and subparticipants.	Sets out a legal and technical framework for cross-border health information exchange and the use of health data across EU member states. It is a binding legislative act which must be applied in its entirety by all EU member states.
Standardization	Builds on common data elements (United States Core Data for Interoperability), a common technical standard for the structured real-time retrieval of discrete data (HL7 FHIR^d^) in addition to conventional document-based queries, and a governance structure to enforce trust and standardization across networks [31,48].	Builds on the EU’s eDelivery Building Block and Public Key Infrastructure, the European Electronic Health Record Exchange Format, HL7 FHIR, and the Digital Imaging and Communications in Medicine standard for the digital storage and transmission of images and related information for interoperability.
Infrastructure	Aims to establish a “network of networks.” Provides the RCE Directory Service and certificate-based trust so data sources can be discovered and exchanged securely at scale	Mandates the setup of not only one but two separate, albeit interconnected infrastructures: MyHealth@EU (primary use) and HealthData@EU (secondary use). Both systems operate a federated network of national nodes.
Data exchange purposes	TEFCA currently defines the following exchange purposes: Treatment, payment, health care operations, public health, government benefits determination, and individual access services. Research has been announced, but has not yet been described or implemented as a valid exchange purpose.	The EHDS does not set out distinct exchange purposes, but it explicitly differentiates between the primary use and secondary use of electronic health data, of which either triggers distinct rights, duties, and interoperability obligations. Primary use is the processing of electronic health data for the purpose of health care delivery, while secondary use includes any other use purposes (eg, public health, research, or education). It also explicitly empowers individuals to access, control, and share their electronic health data and foster a single market for electronic health record systems, supporting both primary and secondary use.
Enforcement	Participation is voluntary. The Common Agreement (in “TEFCA”) constitutes a legally binding agreement between the RCE and a “signatory” that desires to be designated as a Qualified Health Information Network. Moreover, it contains a number of contractual provisions that any Qualified Health Information Network must flow down to its participants and subparticipants. It aligns with federal interoperability goals, and federal agencies may choose to make TEFCA connectivity a requirement or benefit for participating in federal programs like Medicare and Medicaid. With the so-called HTI-2 Final Rule, the Office of the National Coordinator for Health IT introduced a “TEFCA Manner Exception,” meaning complying with TEFCA can help organizations avoid penalties for information blocking, acting as an incentive [49]. Further, TEFCA implicitly requires compliance with the Health Insurance Portability and Accountability Act (HIPAA) of 1996, narrowing gaps between HIPAA-covered and noncovered entities and reducing liability confusion which previously hampered HIE [50].	Establishes enforceable duties on health providers and system vendors, with the European Commission empowered to adopt detailed common specifications to ensure technical convergence. March 2027 has been set as the deadline for the European Commission to adopt several key implementing acts, providing detailed rules for the operationalization of the regulation.

^a^TEFCA: Trusted Exchange Framework and Common Agreement.

^b^EHDS: European Health Data Space.

^c^RCE: Recognized Coordinating Entity.

^d^HL7 FHIR: Health Level 7 Fast Healthcare Interoperability Resources.

In summary, policy efforts globally are rapidly aligning to promote data sharing, reuse, and individual data sovereignty, while implementation must still navigate issues of data protection and sovereignty across different jurisdictions [51,52].

## Large-Scale Data Integration Enables the Creation of DTs

By design (Table 2), both TEFCA and the EHDS Regulation now inherently position EHRs as the primary gateway to the health data ecosystem. Both grant private individuals the right to aggregate their health data from different data holders through any compliant application of their choice. Comprehensive data access and control rights enable private individuals to actively participate in the use of their data and steer data flows, which might also provide an extra incentive for them to donate their data for research.

**Figure 3:**
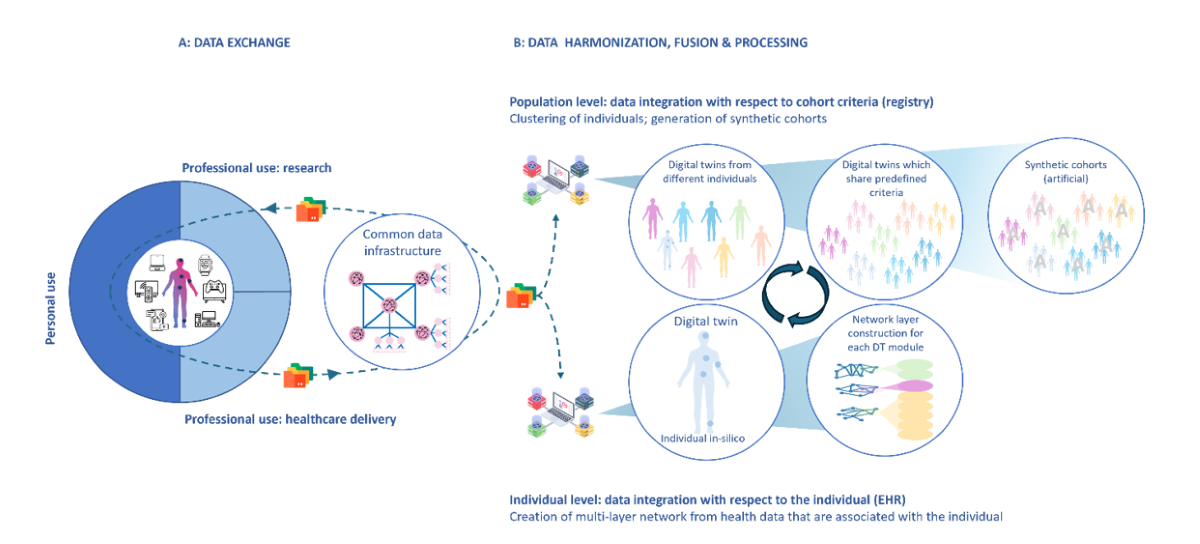
Large-scale data integration and the creation of digital twins. Icons made by vectorsmarket15, Freepik, prettycons, rukanicon, Nuricon, kmg design, Prosymbols Premium, and IconBaandar from Flaticon [32].

When various types of health-related RWD (Figure 1) are combined with reference to the individual, the result is sometimes referred to as medical DT or health DT [29,53]. A DT is a digital representation that mirrors an individual’s health status in real time, constructed from all available data streams [54]. As EHR vendors adapt their systems to keep up with regulatory requirements and stay competitive, the EHR as we know it is likely to evolve into an interconnected data hub. From the perspective of the private individual, the next-generation EHR is set to become their personal data trove. As such, it can be viewed as the “data backbone” for the individual’s DT. Controlled by the individual, it locates and assembles the data that can be fed into mechanistic or statistical models that, for instance, may simulate disease trajectories or predict treatment responses. If such models are directly integrated into EHR software applications, which can be rerun any time new data are added or become known to the EHR (ie, direct data entry into the EHR or reference to updated data in connected sources), the EHR basically becomes the DT of that individual (lower-right part of Figure 3 [32]).

Access to a common data infrastructure enables large-scale data integration. This enables the generation of comprehensive longitudinal EHRs. For instance, all factors that influence a disease can be described by networks (multilayer network construction). Using personal measures from the individual’s EHR as input for these networks creates the individual’s DT [55]. Large-scale data integration across many individuals can then give rise to 2 distinct DT population types: virtual cohorts consisting of the data twins of real people and virtual cohorts of computer-generated “twins,” that is, data from real people is used to generate synthetic data representations of “as-good-as-real” individuals but are not associated with a real living human being. The latter type of cohort, more precisely, is rather a potential application of digital twins that is made possible by leveraging data from real humans to build or improve mechanistic data and process models of different scales and dimensions (eg, organs and organ systems, cellular interactions) with the help of artificial intelligence and use insights gained from these models to create new synthetic data representations [53]. These representations, however, do not constitute actual DTs in strict terms [54-58].

In an LHS, DT cohorts—both “real” and synthetic ones—are invaluable (upper-right part of Figure 3 [32]). The former type of cohorts allows researchers and clinicians to study real-world complexity, diversity, and longitudinal care patterns, while the latter is particularly useful for drug development, for example, when recruitment of a sufficiently large number of real patients is difficult [59]. Marrying the DT concept with population health, one can imagine continuously learning “virtual trials” or simulations that update as new data streams in. This is a sharp departure from the static, periodic data capture of traditional registries.

In traditional registries, data are collected at set intervals or only during certain health care encounters and often with a narrow scope defined by the registry protocol. In contrast, a DT approach can incorporate layers of data (clinical, behavioral, environmental, etc) in near real time [29,54-56,59,60]. The ultimate goal, as some authors have articulated, is to move from surveillance to improving clinical care in real time by integrating these rich data layers in a big data health information system ([1,61,62]; ie, an LHS).

If every individual can decide at any time to make their data accessible for analyses on demand, the registry as a separate entity may not need to collect data in the traditional sense anymore. Instead, it would be the registry’s job to assemble relevant data from the network upon provision of a cohort’s definition. Instead of establishing a new registry and recruiting patients into it, researchers could simply obtain consent from patients to query their DT’s data through the network for specific analyses using the registry’s services if individuals have agreed to be contacted for this purpose. Likewise, individuals should be able to discover trials or studies fitting their individual profile via their EHR interface, making use of the common infrastructure to locate potential matches in connected registries.

Essentially, next-generation registries need to have a software architecture that allows researchers to aggregate data on the fly via queries to a distributed network and contact individuals if needed, for instance, to ask them to provide extra data or invite them to participate in a clinical trial. For example, rather than maintaining a dedicated registry for a disease like multiple sclerosis (MS) and asking clinicians to enter data into it, a future MS registry should be able to identify all patients with MS across EHRs (using standardized definitions and codes) and then retrieve their pertinent data (laboratory data, multiomics, functional test data, imaging data, etc) from various connected sources (digital health apps, insurance claims and billing datasets, medical records, etc) as allowed. On top of this, machine learning models could be used to generate predictive insights (eg, disease progression forecasts) from these integrated datasets, which then could be fed back into care via clinical decision support. In fact, several pilot projects have already explored DTs of various types (eg, DTs of organs such as the lung, the heart, and the spine; DTs of diseases such as Alzheimer or type 2 diabetes) [55,56]. Such models thrive on integrating multimodal data, reinforcing the need for interoperability and propelling the uptake of regulatory initiatives such as TEFCA or the EHDS regulation.

The implementation of these initiatives flips the traditional script: private individuals control their data and decide how to share it, and professional stakeholders assemble the data dynamically as needed and permitted to conduct analyses for their own use or as a service for other stakeholders (including private consumers). The ongoing evolution from a fragmented landscape of siloed information systems to interconnected health data ecosystems represents a paradigm shift in how we approach clinical research, public health surveillance, and health care delivery.

## Opportunities in Interconnected Health Data Ecosystems

### Individual Data Access and Control

Private individuals are gaining the technical means and legal rights to access and consolidate their health information, effectively dissolving the historical monopoly that professional data stakeholders have held over data access [50,62]. This democratization of data could catalyze greater patient engagement in care and research while spurring innovation in digital health technologies that harness comprehensive data. Community-driven data-sharing platforms such as PatientsLikeMe showcase the potential.

### Better Care and Workload Relief

Large-scale data integration can provide more immediate insights for decision support. If physicians can, for instance, query the connected network infrastructure through EHR systems and instantly establish a repository of pooled data to see how similar patients have fared on a certain therapy, they can efficiently leverage knowledge gained from population analyses at the point of care. In this regard, the transition to interconnected health data ecosystems is the prerequisite for achieving the LHS vision where “the process of discovery [is] a natural outgrowth of patient care” [41]. And if data can be reused effectively such that reentry of data is avoided wherever possible, this also means that quality improvement initiatives and safety surveillance can happen in shorter time intervals than before.

### Value-Based Care

A significant driver behind large-scale data integration is the global shift toward value-based care (VBC) [63,64]. Unlike volume-based care (fee-for-service models that reward quantity of services), VBC ties reimbursement and incentives to outcomes and cost-efficiency, essentially rewarding the “value” (quality of care in terms of health outcomes) delivered to patients [65-67]. The implementation of VBC requires a shared definition of health outcomes such that qualitative differences in one and the same outcome can be quantified and compared across providers. Michael Porter, who pioneered the VBC concept, emphasized that data must be “organized around the patient, not the department,” and that all information involved in the care cycle should be transparent and accessible to all stakeholders involved in that patient’s care to drive competition for the best care in terms of value [67]. VBC programs increasingly recognize the importance of patient-reported outcomes and experiences as part of the value equation [68]. These often come from outside traditional clinical data sources, via surveys or apps. Integrating such data with clinical outcomes requires flexible data collection mechanisms that transcend siloed systems. The LHS approach dovetails with VBC when continuous feedback loops are implemented to contain rising health care costs in aging societies [69]: as data (including health outcomes and costs incurred in obtaining them) are collected and analyzed, insights can drive changes in practice to improve value, which are then measured in subsequent data, further incentivizing competition for the best value.

### Population Health Management

Health care providers are increasingly responsible for managing the health of defined populations under accountable care models. This requires identifying high-risk patients, gaps in care, and social determinants of health—tasks that benefit from pooling data from different sources. Traditional registries might tell you about disease-specific outcomes, but large-scale HIE and data integration can provide a panoramic overview, enabling proactive interventions. For instance, it might reveal that certain neighborhoods have higher rates of emergency visits for asthma, correlating with environmental data.

### Health Care Policy Setting

The push for VBC has also spurred multistakeholder engagement in defining what outcomes matter and how to measure them. A recent report by the World Health Organization underscored that many countries are moving to formally involve patients and citizens in valuing care and outcomes [68]. The concept of value is now being extended beyond clinical measures to include aspects like equity and patient experience [15,27,70], and capturing these again requires tapping into various data sources.

## Challenges in Interconnected Health Data Ecosystems

### Data Quality and Bias

When integrating diverse data, especially RWD, we must confront what has been called the “big data paradox” [71]. With massive sample sizes, even tiny biases or errors can lead to statistically significant—but misleading—results [72]. Common issues include missing data, inconsistent data entry, and documentation biases. Additionally, the absence of evidence in a dataset is not evidence of absence (a condition not documented is not necessarily not present). Advanced data cleaning, provenance tracking, and bias analysis techniques will be needed. The process of data collection itself always has an effect on the resulting dataset. Data recording in the process of health care delivery may be professionally supervised, but supervision itself is still subject to differing conditions (eg, organizational workflows, experience of the supervising professional, and reimbursement policies). It has also been noted that data recorded in the process of health care delivery not only reflect the health of a patient, but also the patient’s interaction with the health system [73]. Professional supervision of data collection alone is no guarantee for high quality. It may as well introduce additional data biases through the underreporting of adverse events [74] or the false application of diagnosis-related groups codes to receive higher reimbursements [75]. Data collection led by researchers, too, may be prone to biased selection of study participants, misclassification of individuals with regard to their exposures, outcomes, or covariates, or confounding if no adequate safeguards are taken [76]. Smart design choices in digital health applications, however, can help mitigate errors right at the point of data entry.

### Governance and Privacy

In a traditional registry, governance is typically clear: a registry custodian (often a professional society or public health body) has authority over data use and access according to a protocol. In an open network like TEFCA, governance is distributed. Compliance and enforcement across many networks and their participants are challenging. Data sharing at this scale also heightens concerns about breaches or misuse. Once many systems interconnect, the attack surface expands [50]. Robust security infrastructure, continuous monitoring, and clear accountability when violations occur are essential to maintain trust in the network. The EHDS tries to mitigate that through the establishment of a separate infrastructure for secondary data use and the creation of health data access bodies that act as gatekeepers to that infrastructure.

### Transparency and Individual Responsibility

Consent choices need to be presented in a transparent and understandable manner. Standards guiding the implementation of granular consent options in app interfaces [51] and novel technologies such as blockchain encryption need to be explored [77]. Individuals need to be aware of who may access their data and for what purpose. There is also a need for public engagement and communication about the benefits of data sharing. If patients fear their data will be used against them (eg, by insurance companies or employers), they may opt out of sharing, which could reintroduce biases and silos.

### Interoperability and Standardization Hurdles

Different health data stakeholders still use varying standards or versions of standards. Fast Healthcare Interoperability Resources (FHIR) is gaining traction, but not all legacy systems support it fully. Semantic interoperability (ensuring that a “hypertension” diagnosis means the same in all systems, or that units of measure are standardized) is an ongoing effort. Expanding the breadth of standardized data will be an iterative process. Moreover, not all data types are equally ready for exchange. Structured tabular data are easier to integrate than unstructured notes or medical images. As we integrate more data from different modalities, new standards and mapping efforts will be needed. In recognition that technology is constantly evolving and that standardization needs to be harmonized, TEFCA will be updated over time, iteratively incorporating consented recommendations from representatives from health data stakeholder groups. The EHDS Regulation similarly mandates the setup of a stakeholder forum and includes a step-by-step timeline for operationalization toward full implementation.

### Cultural and Organizational Change

In the past, health data stakeholders have engaged in health data blocking due to commercial protection reasons or liability concerns [36,63,78]. Despite all looming opportunities, professional stakeholders might be wary of patients accessing full records or skeptical of external data quality. There may be resistance to relying on networked data services for research when people are used to the graphical interface and control mechanisms of a dedicated registry. Overcoming resistance to change requires, for example, showing clinicians that using networked data services saves them time, or showing researchers that they can achieve high-quality results faster using such services than by manually assembling data from selected sources. Ensuring that those who traditionally ran registries (often specialty societies or public health departments) still have a vital role—perhaps as stewards of data quality, analytic expertise, or conveners of stakeholders—may also help to reduce resistance.

### Data Noise

Traditional registries, by their focused nature, were good at homing in on a specific problem (eg, tracking all patients with a rare disease to understand its course). An interconnected health data ecosystem provides access to a large pool of data, but analysts will need new methodologies and possibly new tools to ensure that, in the vast sea of data, the critical signals (safety issues, treatment effects, etc) can be detected and acted on.

## Discussion

### Overview

Interoperability initiatives such as TEFCA and the EHDS Regulation signal a globally converging willingness among regulators and policymakers to actively promote HIE among health data stakeholders through the establishment of common rules and standards and the creation of a common technical infrastructure for data sharing.

Taking into account the growing participation in TEFCA within the United States as more Qualified Health Information Networks are designated and the gradual implementation of the EHDS across EU member states, EHR systems are poised to become the major interface through which individuals manage and track their health and encounters with health professionals, gradually turning EHRs into interconnected data hubs. Similarly, we expect that registries will turn into larger IT platforms, equipped with functionalities and a software architecture that enable data pooling and integration from a variety of data sources across a federated data network.

Even though traditional registries failing to adapt might risk becoming obsolete, this should not be misinterpreted as saying that the goals they served are obsolete. On the contrary, common goals—such as understanding diseases, evaluating interventions, monitoring safety, and improving quality—are more important than ever. It is the means of achieving those goals that is changing.

Labkoff et al [17] have identified 3 drivers of change with regard to registries: agility—the demand for systems that can be used for multiple use cases; extreme longitudinality—the demand for systems that allow data tracking over more extended periods of time and with greater frequency; and multimodality—the demand for systems that can ingest multiple data types [17].

Put differently, next-generation registries are the ones that strive to cater to all 3 of these demands. A 3D matrix can also be used to determine how well a certain registry is able to handle complexity along these 3 dimensions relative to other registries (for an exemplary comparison of various existing registries, see [17]).

It is also important to recognize that progress along these dimensions will occur at different paces in different contexts, which is also reflected in the step-by-step rollout of EHDS and TEFCA. Some specialized registries in niche domains or low-resource settings may continue to exist separately for some time, especially if the infrastructure or trust needed for integration is not yet in place. During the transition, hybrid models will persist (eg, registries not yet ready to connect to a common infrastructure but are already capable of pulling/pushing data over designated point-to-point connections). Traditional registries will not disappear overnight but will gradually change or be replaced. We imagine a timeline where more and more registries migrate to pooling data across federated network infrastructures, up to the point when starting a registry “from scratch” without leveraging existing data networks becomes a rarity.

The international alignment of policies, rules, and regulations will still require more work and time to fully exploit arising opportunities and overcome accompanying challenges. International organizations and standards bodies play a crucial role in filtering stakeholder voices and finding universal solutions to common problems, which promotes the development of interoperability standards [48,79]. A comprehensive overview and categorization of relevant interoperability standards can be found in Torab-Miandoab et al [80].

In the literature, several levels of interoperability have been defined, but semantic interoperability can be considered the most important one as it ensures that 2 or more systems are able to exchange information with one another and interpret it correctly [79]. This requires the use of standardized terminology, which enables systems to literally speak the same language, using the same vocabulary [80]. The accurate mapping of local terms to a specific semantic standard, such as Systematized Nomenclature of Medicine–Clinical Terms (SNOMED CT), however, is labor-intensive and demands expertise. The diversity of human languages that are spoken around the world, along with domain-specific vocabulary and local peculiarities, poses significant challenges in the implementation of such standards [81]. Different approaches to tackle these challenges have already been proposed and applied with varying success, for instance, the development and application of guidelines for the adoption of SNOMED CT, or the use of large language models in the mapping process [81,82].

While semantic standards define the meaning of data, transport standards define the structure and format of data that is needed for mutual data exchange. They ensure that data can actually be sent and received [80]. Without transport standards, data cannot actually move between systems. Building common infrastructures is not possible without implementing corresponding standards, which is why they play a prominent role in the implementation of TEFCA and the EHDS Regulation.

Even though the EHDS Regulation does not explicitly mandate the use of Health Level 7 (HL7) FHIR, it is still globally considered one of the most important transport standards for the electronic exchange of health data [80], and HL7 Europe has also published 3 FHIR Implementation Guides especially designed to support the EHDS [83]. In contrast to the EHDS regulation, TEFCA even directly incorporates it as a key standard. Despite its popularity, many HL7 FHIR resources, however, have not yet reached normative status. This means that many HL7 FHIR resources are still in development and undergoing iterative testing. This implies that their definitions are still subject to change or can even be removed in future versions of the FHIR specification. In the current FHIR specification (version 5.0.0.0), only 15 out of 162 resources are listed as normative [84]. On one hand, this may complicate the implementation of FHIR in practice. On the other hand, this also allows for more flexibility in its adoption and leaves room for adjustments to changes brought about by new technologies and accompanying changes in user needs.

Ultimately, many countries are tackling similar issues [35-37]. There is a need for interoperability across borders, especially for research on rare diseases and global health threats. This also highlights the need for joint international initiatives that enable the pooling of resources and provide a forum for discussions and shared decision-making to overcome these issues. The implementation of EHDS, TEFCA, or the Global Initiative on Digital Health by the World Health Organization demonstrates that this has been widely recognized, but implementation is still in the early stages.

This is also a major limitation of the views and ideas we have presented in this article. Future research will be needed to evaluate the real-world impact of these initiatives. Similarly, more research into the design and setup of registries that make use of today’s technological advancements and keep up with user demands in terms of functionalities is needed. The multidimensional rise in complexity of today’s user demands may even justify the formalization of “Registry Science” as a new discipline [17].

### Conclusion

In this viewpoint, we have aimed to describe the ongoing transformation of global health data ecosystems, considering recent global changes in associated legislation and regulations, which promote a shift toward cross-network HIE of unprecedented scale. For this purpose, we have reviewed ongoing discussions in the literature touching on relevant topics and findings concerning, for instance, the use of information systems in health care and clinical research in general and the use of EHRs and registries in particular; the meaning of digitization vs digitalization; contents of TEFCA and the EHDS Regulation; and the application of the DT concept in the sphere of health. In doing so, we have come to the conclusion that up until now, ongoing discussions have largely failed to acknowledge the inherent transformational power of TEFCA and EHDS concerning present health data ecosystems, the role that EHRs and registries have come to play therein, and, closely connected to that, the relevance of these measures for the wider application of DTs in health care and the establishment of LHSs. With the publication of this viewpoint, while connecting the dots between relevant topics, we aim to close this gap and hope to contribute a new angle to the general scientific discussion of registry use cases and the raison d’être of registries in general. In conclusion, we believe that unless registries evolve to embrace embedded, standards-based data services, operating across interoperable infrastructures, they will become obsolete while digitalization is reshaping how data can be collected, shared, and used.

When it comes to the implementation of recent regulatory initiatives and the rethinking of the role of registries as depicted in this viewpoint, however, we must measure the success not by technology adoption alone, but by outcomes. Does integrating data lead to faster discovery of effective treatments? Does it help eliminate redundant tests and procedures? Does it reduce the burden on patients who previously had to enroll in multiple registries or carry their records from doctor to doctor? Early signs are positive. For instance, integrated data networks have been used to rapidly study medication safety signals by aggregating EHR data from many sites, something that would have taken traditional registries years to accumulate [85]. There is also the possibility that large-scale data integration can enhance HTAs and surveillance by providing a more complete picture than any single data source could [10,11,86]. To succeed, professional health data stakeholders need to further adopt common data models and application processing interface standards such as FHIR, support harmonization efforts, and dedicate more attention to the role of private individuals as major owners and donors of data. This, for instance, includes patient involvement in the creation of EHR applications and user-centered design of feedback loops in any health data applications. Private individuals, on the other hand, need not only to be made aware of their rights to data access and control, but must also make use of the opportunity, take action, and use those rights.

## Abbreviations

DT: digital twin
EHDS: European Health Data Space
EHR: electronic health record
FHIR: Fast Healthcare Interoperability Resources
HIE: health information exchange
HL7: Health Level 7
HTA: health technology assessment
LHS: learning health system
MS: multiple sclerosis
RWD: real-world data
SNOMED CT: Systematized Nomenclature of Medicine–Clinical Terms
TEFCA: Trusted Exchange Framework and Common Agreement
VBC: value-based care
